# Correlated Light-Serial Scanning Electron Microscopy (CoLSSEM) for ultrastructural visualization of single neurons *in vivo*

**DOI:** 10.1038/s41598-018-32820-5

**Published:** 2018-09-27

**Authors:** Yusuke Hirabayashi, Juan Carlos Tapia, Franck Polleux

**Affiliations:** 1 Columbia University Medical Center, Department of Neuroscience, New York, USA; 2 Mortimer B. Zuckerman Mind Brain Behavior Institute, New York, USA; 3 Kavli Institute for Brain Science, New York, USA; 4 JST, PRESTO, Kawaguchi-shi, Japan; 5 University of Talca, Faculty of Health Sciences, Talca, Chile

## Abstract

A challenging aspect of neuroscience revolves around mapping the synaptic connections within neural circuits (connectomics) over scales spanning several orders of magnitude (nanometers to meters). Despite significant improvements in serial section electron microscopy (SSEM) technologies, several major roadblocks have impaired its general applicability to mammalian neural circuits. In the present study, we introduce a new approach that circumvents some of these roadblocks by adapting a genetically-encoded ascorbate peroxidase (APEX2) as a fusion protein to a membrane-targeted fluorescent reporter (CAAX-Venus), and introduce it in single pyramidal neurons *in vivo* using extremely sparse *in utero* cortical electroporation. This approach allows us to perform Correlated Light-SSEM (CoLSSEM), a variant of Correlated Light-EM (CLEM), on individual neurons, reconstructing their dendritic and axonal arborization in a targeted way via combination of high-resolution confocal microscopy, and subsequent imaging of its ultrastructural features and synaptic connections with ATUM-SEM (automated tape-collecting ultramicrotome - scanning electron microscopy) technology. Our method significantly will improve the feasibility of large-scale reconstructions of neurons within a circuit, and permits the description of some ultrastructural features of identified neurons with their functional and/or structural connectivity, one of the main goal of connectomics.

## Introduction

Unbiased-saturated connectomic approaches are aimed at reconstructing all synaptic connections within neural circuits^1–3^. Despite significant technological advances, current approaches cannot be generally applied to the description of large-scale neuronal connectivity at ultrastructural resolutions because of the following major roadblocks^4^. Current SSEM approaches are mainly applied to small pieces of brain tissue (~hundreds of cubic microns), which is incompatible with the mapping of long-range connections organized over millimeters to meters^5^. Within a given volume, the identity of each of the elements in the circuit (cell types, axons, dendrites, synapses) is undetermined and there are limited ways to identify the axons or dendrites originating outside the volume reconstructed by SSEM.

Fluorescent light microscopy (LM) however, coupled to genetic labeling of neurons, allows the identification and tracking of axons, dendrites and their branches over long distances in fixed and intact behaving animals. Unfortunately, this approach only informs about target regions, giving no information about synaptic partners. Therefore, the correlation of LM to electron microscopy-imaged structures (CLEM) could overcome the problems associated with the study of long-range circuits^6^. Although many efforts have been performed in this direction, a prevailing limitation of most CLEM studies is that the tools used to label specific cells obscure most of their ultrastructural features severely affecting their implementation^7–9^. There is an urgent need to characterize single neurons at the molecular, cellular and circuit levels. Because individual synapses have dimensions below the light diffraction limit, neuronal circuit reconstructions at ultrastructural levels require the use of either super-resolution microscopy or serial section electron microscopy (ssEM). The advantage of EM approaches over super-resolution microscopy is that it provides rich and unbiased ultrastructural information of synapses as well as details about the organelles and subcellular elements that compose individual neurons^10,11^. Several recent studies have attempted to correlate the functional and structural properties of neuronal ensembles defined by fluorescent imaging with the mapping of their synaptic connections using serial EM^12,13^. However, several roadblocks limit the general applicability of such approaches to the mammalian central nervous system (CNS): (1) the difficulty of mapping long-range connections over hundreds of microns between genetically- and/or functionally-identified neurons within a circuit, and (2) the fact that most genetically-encoded EM contrasting agents (HRP, APEX, miniSOG) are delivered to the cytoplasm, obscure the neurons ultrastructural information disabling a detailed description of their subcellular features^7,8^, (3) the requirement of large amount of tracing by the experts, or specialized equipment and reagents.

A recently developed monomeric peroxidase reporter, APEX2^14^, which keeps its enzymatic activity even in the reducing cytosolic environment of cells or upon fixation, has been shown to be useful for studying protein localization in cultured cell lines and in zebrafish using electron microscopy^9,15^, but its application in mammalian tissue including the brain has been scarcely explored. Here, we took advantage of ATUM-SEM^10^ to interrogate the ultrastructure of large pieces of tissues (~1–4 mm) and correlate LM and EM datasets obtained from single mouse cortical pyramidal neurons. We demonstrate that by expressing a plasma membrane-targeted APEX2-Venus-CAAX fusion protein, we significantly increase the accuracy and feasibility of performing CLEM *in vivo*, allowing the imaging of single layer 2/3 pyramidal neurons and their synaptic connections while preserving the ability to visualize ultrastructural details.

## Results

**Figure 1:**
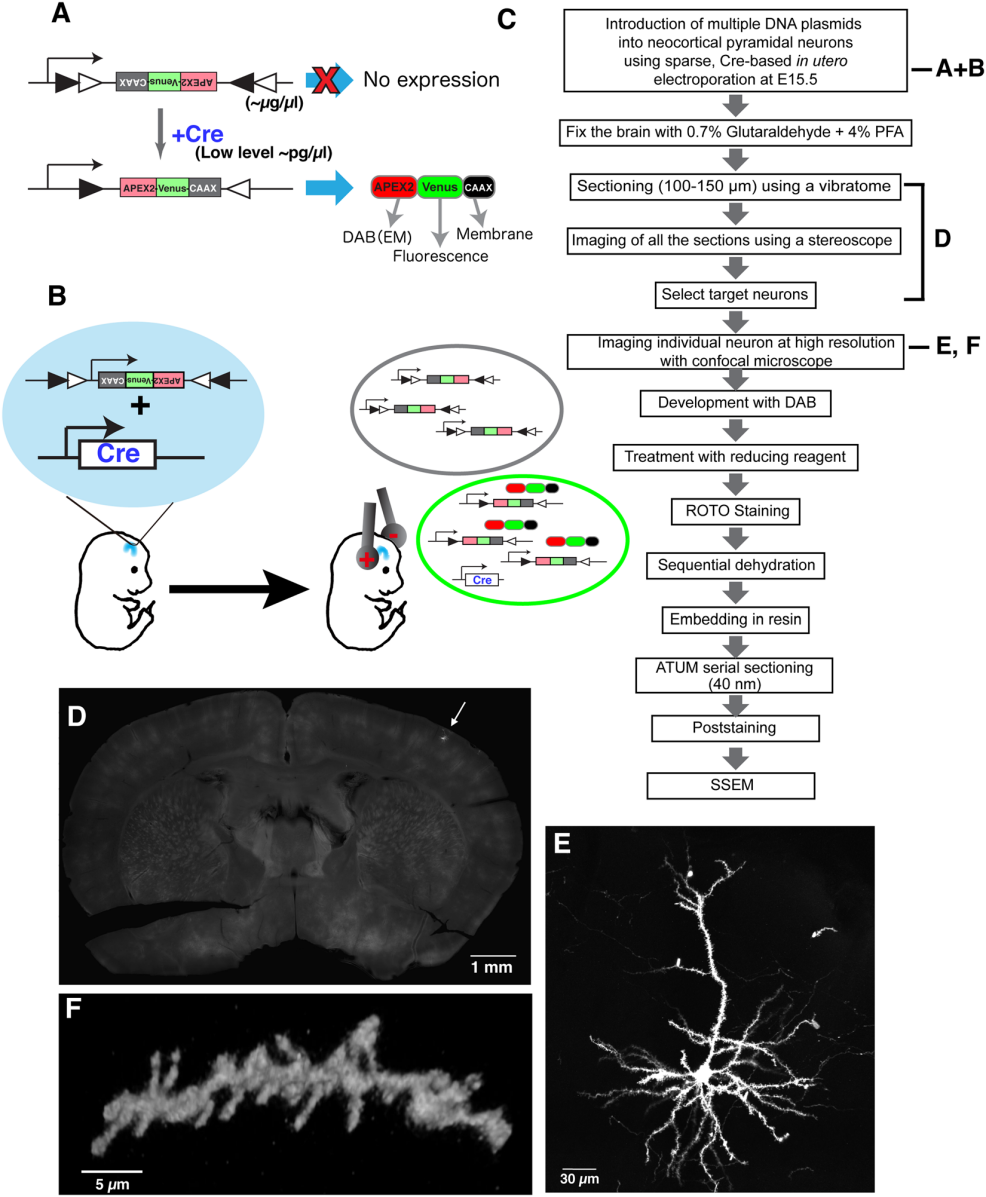
Sparse labeling and fluorescent imaging of individual cortical pyramidal neurons using CAAX-Venus-Apex2 for CoLSSEM. (**A**) Our labeling strategy consists in sparse labeling of single neurons with a Flex plasmid (inverted cDNA flanked by incompatible LoxP sites) which, upon Cre-mediated recombination leads to expression of a fusion protein, APEX2-Venus-CAAX (AVC). DAB, diaminobenzidine. EM, electron microscopy. (**B**) Sparse labeling of individual neocortical pyramidal neurons by *in utero* electroporation. Black circle indicates cells taking up only Flex plasmids. Green circle indicates a cell taking up both Flex and Cre plasmids and expressing APEX2-Venus-CAAX. (**C**) Flow diagram showing the steps involved in CoLSSEM. ROTO; Reduced Osmium Tetroxide-thiocarbohydrazide-Osmium. (**D**) 100 µm thick coronal section of a brain sparsely labeled with Venus (arrow) at postnatal (P) 25. (**E**,**F**) 3D reconstruction from confocal images of a layer 2 cortical neuron at P25 (**E**, 74 z-stack images with 0.9 µm z-step) and a dendritic segment with spines at P23 (**F**, maximum projection of 17 z-stack images with 0.8 um z-step) labeled with AVC fusion protein (also see Supplemental Movie 2 for 3D animation).

In order to improve our ability to perform CLEM studies in mammalian neurons *in vivo*, we decided to design a cDNA that encodes a fusion protein between the fluorescent protein Venus and the recently-engineered plant ascorbate peroxidase (APEX2, Fig. 1A)^14^. APEX2 catalyzes the polymerization and local deposition of diaminobenzidine (DAB) in the presence of H_2_O_2_, which in turn, recruit electron-dense osmium responsible for the EM image contrast (Fig. 1A). Since labeling the cytoplasm dramatically interferes with proper visualization of subcellular structures (mitochondria, ER, synapses), a CAAX motif derived from H-Ras was added to the APEX2-Venus genes to tether the APEX2-Venus-CAAX (AVC) fusion protein to the plasma membrane (Fig. 1A)^16^. We tested the feasibility of the construct primarily in HeLa cells, which after being transfected for 24 hours showed the expected results, only membrane processes were labeled with Venus protein (fluorescence) and visible by light microscopy after DAB processing (data not shown, see Methods for details).

**Figure 2:**
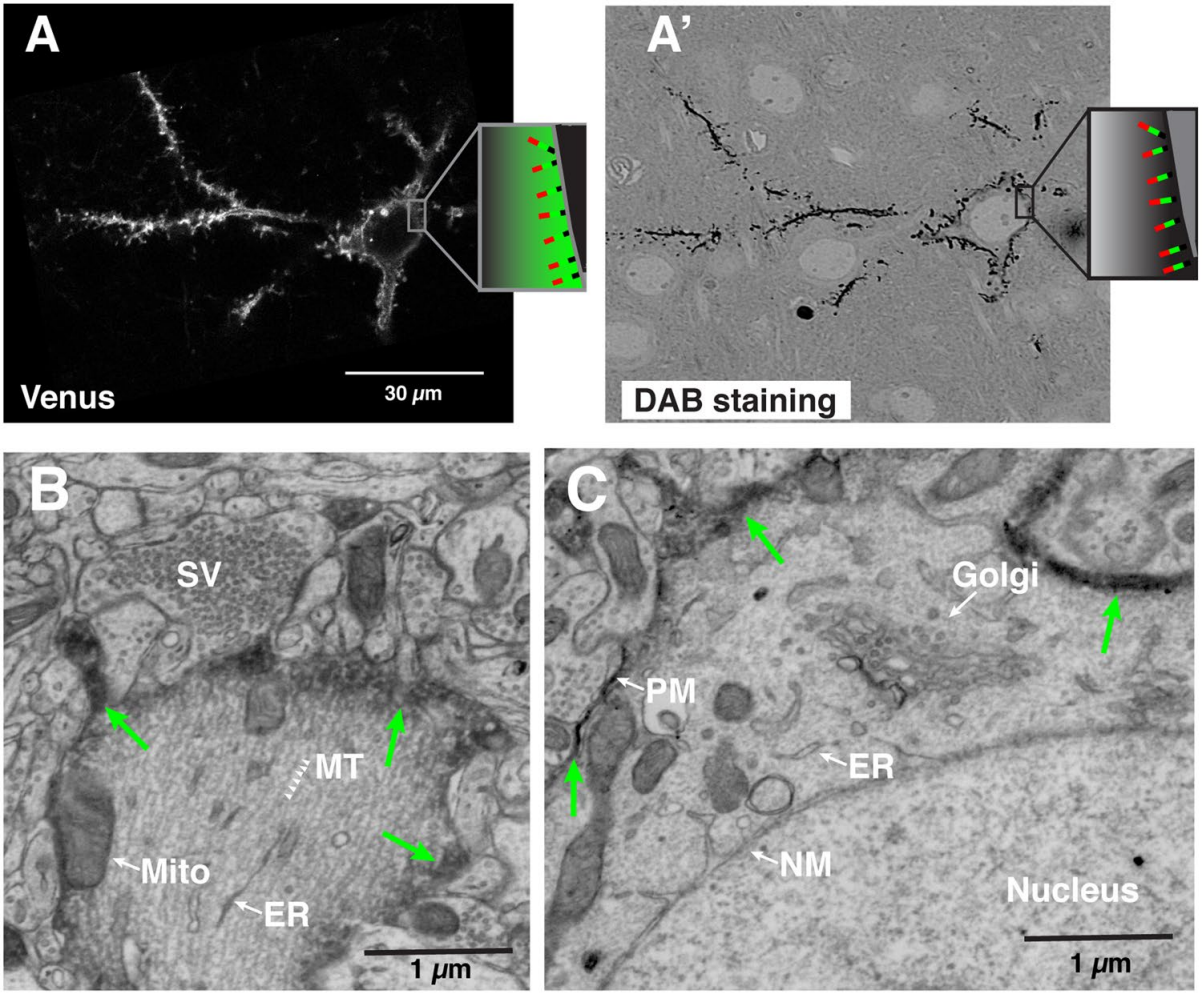
Correlative LM-EM labeling of single neocortical neurons. (**A**,**A’**) Correlated images of Venus imaged by confocal microscopy (**A**) and DAB signal taken by the electron microscope (**A’**) from a single neuron labeled with APEX2-Venus-CAAX protein. (Inset; Schema illustrating the topology of the AVC protein on the plasma membrane; Red, Apex2. Green, Venus. Black, CAAX motif). The thickness of the section imaged in (**A’**) was 1 µm. (**B–D**) Single scanning electron micrographs showing the intracellular structures of a single labeled pyramidal neuron in layer II/III at P25. Green arrows indicate the plasma membrane labeled with APEX2-mediated DAB staining. (**B**) Primary apical dendrite. Arrowheads indicate microtubules (MT). (**C**) Cell body and its resident organelles: the Golgi apparatus, Nuclear membrane (NM), and the plasma membrane (PM) labeled with electron-dense DAB from APEX2. Abbreviations: SV, synaptic vesicle. MT, microtubule. Mito, mitochondria. ER, endoplasmic reticulum.

Although genomic integration of marker genes driven by cell-type specific promoters is a versatile and reliable technique for labeling neurons *in vivo*, the dense labeling of cells in small volume significantly hampers the identification of dendritic and axonal processes from individual neurons. To reduce the number of neurons expressing the AVC fusion protein, without compromising its expression level, we decided to build a plasmid that contained an expression cassette flanked by pairs of incompatible LoxP recombination sites (Flex)-system (Fig. 1A)^17^. The Flex plasmid encoding the AVC gene, and a plasmid encoding the Cre recombinase gene were mixed at 10,000:1 dilution ratio, and injected into the lateral ventricle of a mouse embryo (embryonic day 15.5). The plasmids were introduced into neural progenitor cells in the ventricular zone by using *in utero* cortical electroporation^18–20^. Using such low concentration of Cre-expressing plasmid (~100–200 pg/μl) leads to stochastic recombination of the Flex-AVC plasmid in progenitors of layer 2–3 pyramidal neurons in the ventricular zone of the dorsal telencephalon leading to very sparse expression (Fig. 1B)^21^.

Following long-term survival after *in utero* electroporation, mouse brains were processed for the CLEM approach (Fig. 1C, Methods). Coronal brain slices were obtained using vibratome sectioning (100 microns), and individual optically-isolated Venus-expressing pyramidal neurons in layer 2–3 were identified by imaging all serial vibratome sections from the adult mouse brains, covering the entire somatosensory area, at low magnification with a fluorescent stereoscope (equipped with a X-Y motorized stage; see Methods) (Fig. 1D and Supplemental Fig. 1). Using this approach, the number of neurons expressing the AVC fusion protein varied among individual mouse brains from 10–30 neurons/brain. The cell bodies and processes of the optically isolated pyramidal neurons were then imaged using a confocal microscope, and high resolution image stacks were collected to document the detailed dendritic and axonal morphology at sub-micron resolution (Fig. 1E,F, see also example in Figs 2A and 5A, and Supplemental Movies S1, S2 and S4). Analysis of the spine density in apical oblique dendrites of layer 2/3 neurons at P23 (1.05 ± 0.12 spines/µm, mean ± s.d.) as well as the number of primary dendrites (5.3 ± 1.2 dendrites/cell) indicates that AVC fusion protein is innocuous for these neurons (control cells = 0.90 ± 0.04 spines/μm and approximately 6–7 dendrites per cell)^22,23^. Moreover, this LM approach provided information essential for determining the morphological features of individual layer 2/3 pyramidal neurons as shown in Fig. 1, and to track their long-range axonal projections (see Fig. 5). These results suggest that AVC is distributed to entire neuronal compartments from the dendritic spines to the axon.

Following treatment with DAB and H_2_O_2_, the membrane targeted APEX2 converted the fluorescently tagged neurons (CAAX-Venus) into a dark, electron-dense precipitate (Fig. 2A-A’), compatible with EM detection (discussed below). Sections containing the labeled neurons were processed for ssEM as previously described^10,24^. To align the LM and EM images in the z-dimension, we placed each 100 µm-thick section between two plastic plates and kept the sections flat during the resin embedding process (see Methods). Then resin-embedded thin sections were collected by ATUM in the same plane as the confocal images. The neurons imaged under the LM were easily re-identified in the EM images by visual inspection of darkly outlined somata, allowing correlated light-EM microscopy (Fig. 2A-A’). Whereas the subcellular membrane structures were partially disrupted in the images obtained from the brain fixed with 0.25% glutaraldehyde (GA) and 4% paraformaldehyde (PFA) (data not shown), they were well preserved and DAB staining was optimal when GA concentration was increased to 0.7% (Fig. 2B,C). At higher magnification, it is possible to observe that the DAB staining at the plasma membrane clearly labels the target neuron. And importantly, the labeling does not interfere with the visualization of organelles, cytoplasmic content and nuclear structures in the cell body and dendritic shaft (Fig. 2B,C).

Since the diameter of spine necks range from 100–500 nm^25^, it has been challenging to trace the spines in ssEM studies and in some cases, the imaging and annotation conditions make the connection of the spine heads to their parent dendritic stem nearly impossible (Fig. S2A). In ssEM reconstructions of AVC-labeled neurons however, the spine heads are clearly labeled by the DAB processing, and their tracking from several serial sections to the original DAB-labeled dendritic shaft are clearly identifiable, making the reliability of the ssEM annotation process more accurate and increasing morphological completeness (Fig. S2B-B’). Thus CoLSSEM facilitates disambiguation of fine morphological structures during tracing of neuronal processes.

**Figure 3:**
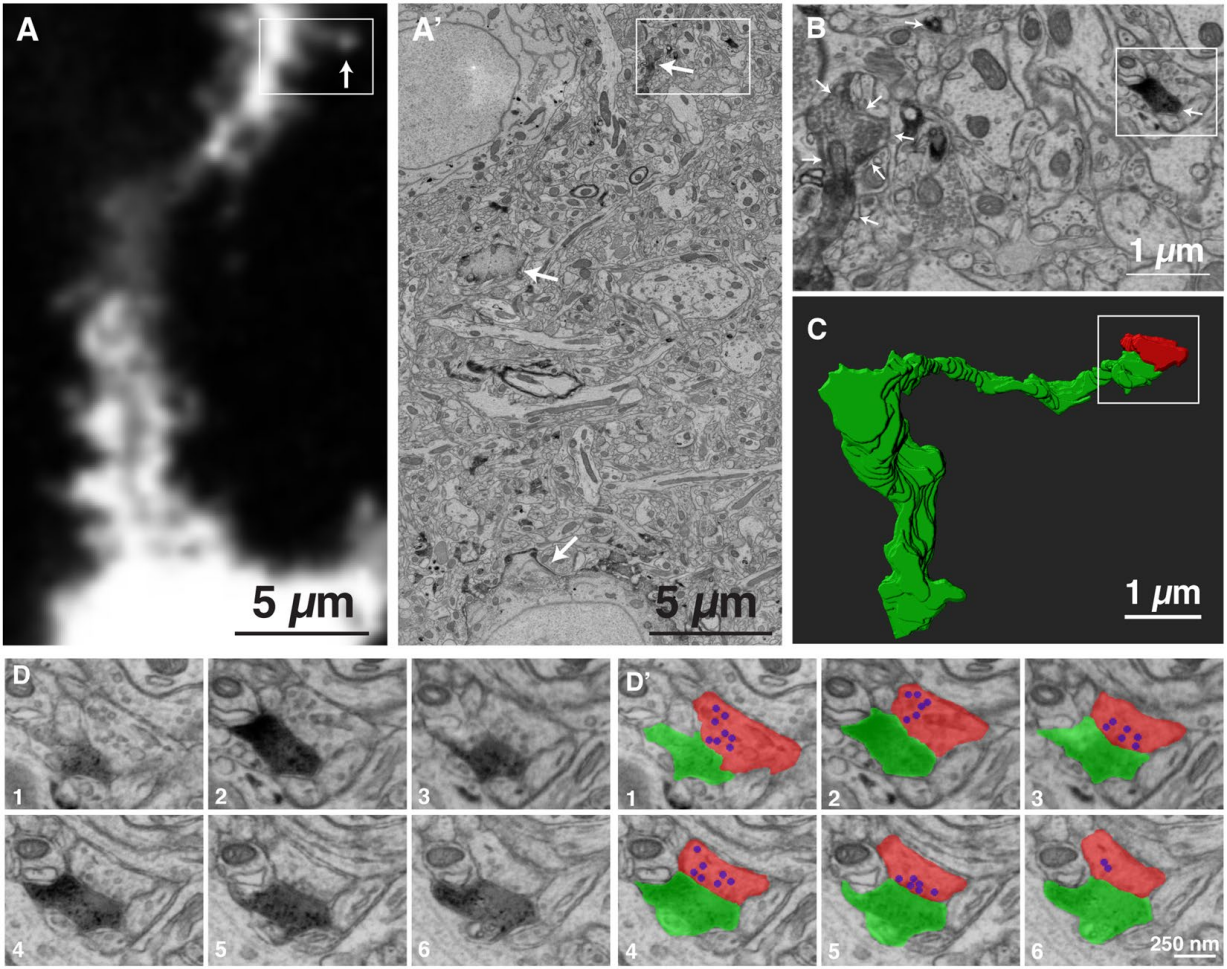
Correlative LM-EM imaging of a dendritic spine and its presynaptic partner. (**A**) A single confocal light microscopy image of a dendrite labeled with AVC. The arrow indicates the spine reconstructed in (**C**,**D**). (**A’**) An electron micrograph corresponding to the area show in (**A**). The area surrounded by a rectangle is shown in (**B**). (**B**) Magnification of the area indicated by a rectangle in (**A’**). The area surrounded by a rectangle is shown in (**D**). Arrows in (**A’**,**B**) indicate AVC labeled neuron. (**C**) The structure of a spine shown in (**A**) reconstructed from serial electron micrograph. Green; labeled spine, Red; its presynaptic partner. (**D**) Serial electron micrograph of the area indicated in (**B**). (**D’**) The DAB staining is highlighted with green and the presynaptic partner of the labeled spine is highlighted with red. Synaptic vesicles in the presynaptic bouton are highlighted with blue.

The drawback of LM is the lack of information about non-labeled cells. Using CoLSSEM, we also revealed the ultrastructural feature of a presynaptic partner (non-labeled) connected to a specific spine (labeled) identified by LM. We identified a dendritic spine by fluorescent microscopy (Fig. 3A), and unambiguously re-identified the structure under the ssEM images by measuring the distance from the cell body and tracing the dendrites (Fig. 3A’,B). By reconstructing the tracing of the spine from the dendrites, the detailed morphology of the spine was clearly visualized (Fig. 3C). In addition, we observed the ultrastructural feature of the presynaptic bouton contacting this spine (Fig. 3D,D’).

**Figure 4:**
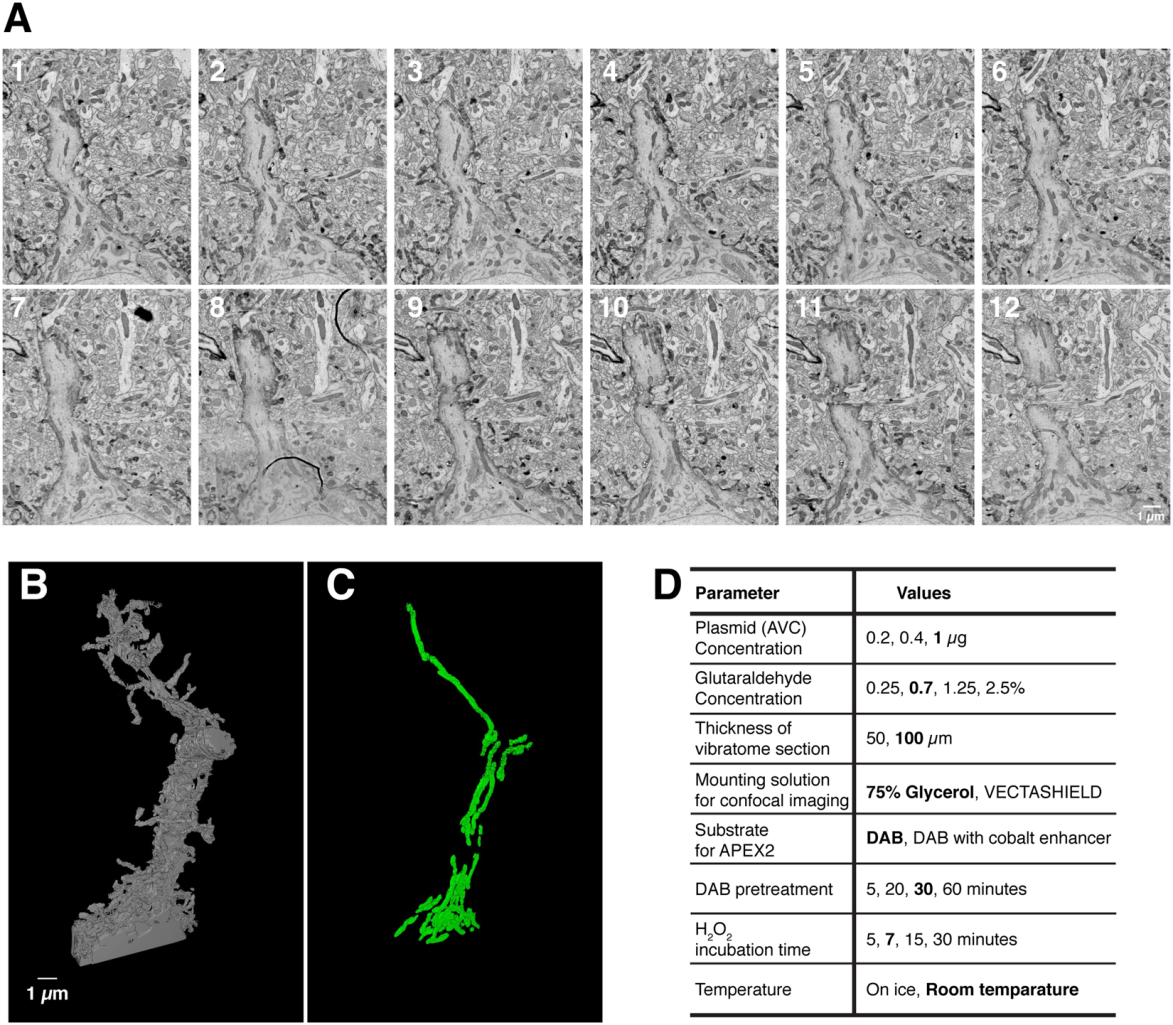
3D-SEM reconstruction of the primary apical dendrite of single AVC-labeled pyramidal neuron and its subcellular features such as mitochondria. (**A**) Montages of serial electron micrographs including the DAB labeled pyramidal neuron shown in Fig. 1D,E. (see Supplemental Movie 3). (**B**,**C**) 3D reconstruction of the plasma membrane (**B**) and the mitochondria (**C**) of its labeled apical dendrite (see Supplemental Movies 4 and 5). To find out the region of interest, 256 serial sections were imaged with 30 nm/px resolution. The region of interest was re-imaged with 3 nm/px resolution through 103 serial sections and reconstructed. (**D**) Parameters tested to optimize the DAB staining and preservation of the membrane structures for SSEM. Bold indicates the optimal conditions used in the paper.

To assess the consistency of the DAB-AVC mediated staining and examine the ultrastructure of an AVC- labeled neuron, we performed the reconstruction of a segment of a labeled dendrite from several ssEM images. Typically, small molecule penetration into glutaraldehyde fixed tissue is a limiting factor^26^. Consistent with this idea, simultaneous incubation of tissue sections with both H_2_O_2_ and DAB substrates for 5–15 min did not give us clear, well-defined membrane labeling. However, treatment of glutaraldehyde fixed vibratome sections with the DAB substrate for 30 min prior to the treatment with H_2_O_2_ significantly improved the DAB-AVC mediated deposition, providing evenly stained neuronal processes throughout the 100 µm section (Fig. 4A, a summary of the optimization Fig. 4D). Then, we reconstructed the apical dendritic trunk from DAB-labeled layer 2/3 neurons by manual tracing (Fig. 4B). Despite the strong staining following DAB pre-incubation, all cytoplasmic organelles such as mitochondria, endoplasmic reticulum (ER) and Golgi apparatus were readily identified (Fig. 4A,C and Supplemental Movie 3).

**Figure 5:**
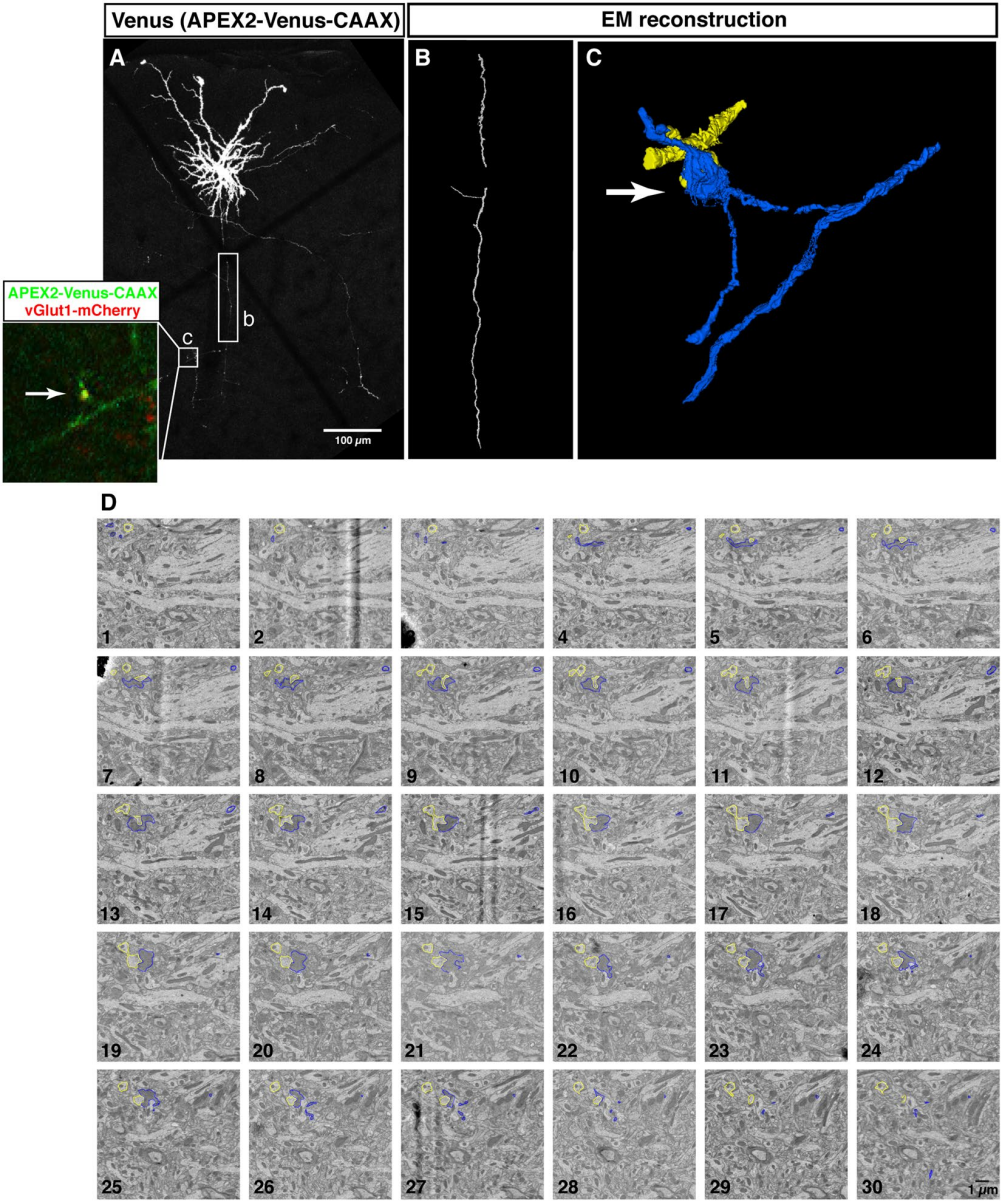
3D correlative imaging of neocortical axons. Confocal images and electron micrographs of a neuron (P28) electroporated with AVC together with vGlut1-mCherry as a presynaptic marker. (**A**) A maximum projection image of a layer 2/3 neuron projecting to layer 5. (Inset) Close-up view of the area indicated by a square (c), which is reconstructed by ssEM in (**C**,**D**). The presynaptic site is labeled with vGlut1-mCherry (red -inset). Rectangle (b), the axonal part reconstructed in (**B**). (**B**) 3D reconstruction of the axon indicated by a rectangle (b) in (**A**). 216 serial sections were imaged with 20 nm/px resolution. The region of interest corresponding to the rectangle (b) was re-imaged with 8 nm/px resolution through 103 serial sections and reconstructed (see Supplemental Movies 6 and 7). (**C**) 3D reconstruction of the labeled axon and presynaptic site (blue) and its postsynaptic target (yellow), corresponding to the region marked by the square (c) in (**A**). The presynaptic bouton was identified by vGlut1-mCherry labeling by LM. (**D**) Montage of electron micrographs corresponding to the square (c) in (**A**). The DAB labeled axon and presynaptic site and its postsynaptic target were manually segmented and highlighted with blue and yellow, respectively.

Tracing axonal segments over long distances from identified neurons would be a major advance for connectomics studies. However, it has been challenging to perform long range axonal reconstructions from ssEM images because axons are long (>hundreds of microns), thin (<1 μm diameter), branch profusely, and adopt tortuous 3 dimensional trajectories. By using the DAB-AVC method, since the axon of interest is well labeled by the DAB staining protocol, compared to the surrounding non transfected neuronal processes, and confirmed by a priori knowledge of the confocal fluorescent imaging, it is possible to follow the axon over more than 100 adjacent ssEM images further away from the cell body. As a result, we could perform correlated light- and SSEM imaging of individual axons, and unambiguously trace a single axon and its branches for >200 µm long or more in the EM images (Fig. 5A,B and Supplemental Movies 4–7).

The challenge represented by tracing a single axon of an identified neuron for a long distance is a major roadblock for identifying presynaptic boutons of a specific neuronal subtype in the SEM images. The correlated imaging of Venus and DAB circumvents this limitation by re-identifying individual presynaptic boutons visualized in the LM images. The presynaptic bouton of layer 2/3 pyramidal neuron in layer 5 was identified by the enrichment of vGlut1-mCherry^27,28^ on the Venus labeled axon in LM (Fig. 5A inset). By correlating this site in the EM sections using DAB staining, the structure of this presynaptic site and its apposed postsynaptic structure were reconstructed from the ssEM imaging (Fig. 5C,D and Supplemental Movie 8).

## Discussion

In this study, we combined a well-established gene-delivery approach to label a single neuron with a novel fusion protein allowing the performance of correlated light- and SSEM imaging (CoLSSEM). The use of conventional two image modalities (LM and EM) grant access to the examination of architectural features on identified neurons as well as the visualization of synaptic connections of specific type of neuron in the mammalian neocortex, without the need for specialized equipment. Our results demonstrate that CoLSSEM improves the accuracy and feasibility of linking multiscale neuronal projections i.e., from long-distance axonal and dendritic profiles (mm^3^) to synaptic connectivity (nm^3^) of a single neuron and close synaptic partners.

By adopting a recently developed peroxidase (APEX2), and targeting it to the plasma membrane as a fusion protein with Venus (Apex2-Venus-CAAX), we have been able to specifically ‘highlight’ the plasma membrane of single neurons from the dendritic spines to the axon *in vivo*. Since enzymatic activity of APEX2 is preserved after treatment with chemical fixatives, the short-term (5–10 minutes) treatment with H_2_O_2_ in presence of DAB substrate permits optimal staining to both highlight a target cell plasma membrane from the surrounding non-labeled cells and maintain intact neuropil complexity. The short-term H_2_O_2_ treatments are beneficial to preserve tissue quality, since long term incubations required for developing staining with HRP (~1.5 h) disrupt significantly the integrity of membranes and neuritic processes. An additional advantage of the AVC approach is that most of somatic, dendritic and axonal organelles remain visible by electron microscopy except for the detailed structures in the spines and fine axons where the DAB-derived Apex2 detection can obscure cytoplasmic content. Further, LM-EM dual labeling from a single protein enabled unambiguous correlation of the neurons between LM and EM. Increased image quality and reliability of light-SSEM correlative imaging helps precise description of ultrastructural features of single cells in complex tissues such as the brain.

However, the approach described in this study also has some limitations that will need to be improved for future applications. The main one is that despite being targeted to the plasma membrane, the AVC genetically-encoded reporter generates electron dense precipitates following DAB reaction that can diffuse in the cytoplasm and obscure some ultrastructural features in structures characterized by very small cytoplasmic volumes (~hundreds of nm^3^) such as axons and some dendritic spines. Despite this limitation, combining genetically encoded fluorescent synaptic marker (such as presynaptic marker vGlut1-mChrry in Fig. 5) with AVC allows for fluorescent confocal imaging providing single synapse resolution and subsequent ssEM reconstruction of the postsynaptic partner of this presynaptic bouton identified by light microscopy. This type of correlated LM-EM approach should enable identification of synaptic organization within genetically tractable cell types within mammalian circuits.

Future applications of our approach will need to test the use of Flex-AVC probe and express it in transgenic mice expressing Cre recombinase in genetically identified cell classes within the developing and adult brain. The rapid development of new mouse lines expressing Cre in a cell-type specific way will allow sparse or dense labeling of ensembles of neurons and perform mapping of the synaptic connectivity within circuits at ultrastructural resolution^29–32^.

In addition to the labeling by genetically encoded probes, the combination of AVC sparse labeling and ATUM-SEM significantly facilitates the efficiency of translating light information into ultrastructural data, and thus easy re-identification of the labeled neurons. By titrating the gene delivery approach adopted in this study, we have been able to label neurons sparsely enough to unambiguously trace their projections across hundreds of microns, which improves the correlation of fine processes at the LM and EM level. The combination of fast, low-resolution fluorescence imaging with the ATUM-SEM approach enabled us to pre-scan hundreds of serial sections in regions that included the cell soma, and areas further away that exclusively contained the AVC labeled neuronal processes. We foresee, the CoLSSEM approach as a significant methodological step toward improvement of long-range single axon imaging^33^, since it significantly improves processing, targeting and acquisition speed of CLEM datasets. In addition, combination of the current CoLSSEM approach with other techniques, such as multicolor electron microscopy and darker staining of PSD with heavy metals, will facilitate more precise examination of the ultrastructural features.

Besides facilitating LM and EM studies, the sparse labeling of neurons with the AVC probe will improve connectomic approaches. We traced the axon from layer 2/3 neocortical neurons for more than 200 µm, which is longer than most tracing studies using ATUM-SEM^34,35^. The membrane contrast provided by our membrane-targeted APEX2 labeling strategy could benefit automated segmentation and tracing of neuronal processes, a critical step for the emergence of large scale connectomics^36^. Given that our LM-EM dual labeling technique can be applied to any cell types by expressing Cre gene from cell type specific promoters, CoLSSEM will improve the description of the ultrastructural features and the connectivity characterizing specific cell types within neural circuits *in vivo*. The ability to combine this strategy with other approaches (*in vivo* morphological and functional imaging) will provide critical new insights into the relationship between neuronal properties, circuit connectivity and its emerging functional properties underlying complex behaviors such memory formation or sensory perception and cognition.

## Methods

### Animals

All animals were housed and handled according to protocols approved by the Institutional Animal Care and Use Committee (IACUC) at Columbia University. Time-pregnant CD1 females were purchased from Charles River (Wilmington MA, USA).

### DNA constructs

The pCAG-vGlut1-mCherry and pCAG-Cre vectors were previously described^37^. The pcDNA3 APEX2-NES (Addgene plasmid #49386) and pAAV-Ef1a-DIO eNpHR 3.0-EYFP (Addgene plasmid #26966) vectors were gifts from Drs. Alice Ting (Stanford University) and Karl Deisseroth (Stanford University), respectively. The flag-APEX2-NES sequence was cloned into the 5′ end of the Venus sequence and the CAAX Motif-AAGCTGAACCCTCCTGATGAGAGTGGCCCCGGCTGCATGAGCTGCAAGTGTGTGCTCTCCTGA (KLNPPDESGPGCMSCKCVLS) was fused to the 3′ end of the Venus cDNA (APEX2-Venus-CAAX, AVC). Then, APEX2-Venus-CAAX (AVC) sequence was exchanged with the eNpHR-EYFP segment of the pAAV-Ef1a-DIO eNpHR 3.0-EYFP vector by AscI-NheI sites (pAAV-Ef1a-DIO-AVC).

### Introduction of DNA into mouse neocortex

The in utero electroporation method was performed as previously described^38^. Briefly, a mixture of the vectors pAAV-EF1a-DIO-AVC (Flex-AVC; 1 µg/µl) and pCAG-Cre (recombinase; 100–200 pg/µl) was injected into the neocortical lateral horn of embryos obtained from a timed-pregnant CD1 mouse female at embryonic day 15.5 with Picospritzer III (Parker, Hollis NH, USA). For co-labeling neuronal processes and their presynaptic sites (Fig. 4), The pCAG-vGlut1-mcherry (0.3 µg/µl) were added to the mixture of the vectors indicated above. The cDNA mixtures were then electroporated into the neural progenitor cells resided in the ventricular zone by 5 electric pulses (50 ms) of 38 V using Tweezer-type platinum electrode (diameter 3 mm; NEPA GENE) and an electroporation system ECM 830 (BTX, Holliston MA, USA).

### APEX development and light microscopy imaging of brain sections

Following anesthesia, mice were heart perfused with 5 ml of phosphate buffered saline (PBS 0.1 M) and subsequently with 40 ml of the fixative solution containing 4% paraformaldehyde (EM grade, EMS) and 0.25–0.7% of Glutaraldehyde (GA, EMS) diluted in PBS. Brains were then dissected out and post-fixed in the same fixative solution for 3 hours. After embedded in low melting agarose (3%, MP Biomedicals), brains were sectioned at 100 µm thickness with a Leica VT1200S vibratome. Sections were placed on glass slides, mounted in PBS, and covered with coverslips. The series of thick sections were first imaged at low resolution (2.1 µm/px, exposure 1 s) with a 1x (0.15 NA) objective on an automated SMZ18 stereoscope (Nikon) equipped with a fast ORCA Flash 4.0 (Hamamatsu Photonics) and a motorized x-y stage (Prior). Following this process, sections containing individually brightly labeled neurons were remounted in 75% glycerol-PBS with coverslips, sealed with nail polish and imaged at higher resolution with either a 20x (0.75 NA) air objective or 60x (1.4 NA) oil objective on a Nikon Ti-e inverted microscope equipped with a Nikon A1 confocal. Sections were washed (PBS for 5 min) and immersed in a PBS containing the APEX substrate (ImmPact^TM^ DAB Chromogen; Vector) for 30 minutes followed by treatment with ImmPact^TM^ diluent (Vector) for 7 minutes. The strongly APEX-developed sections were washed with PBS (3 times) and treated with 10 mg/ml the reducing agent Sodium HydroSulfate (Sigma) in PBS for 10 minutes as indicated^9^. Sections were washed 3 times with PBS, and post-fixed with 2.5% GA overnight at 4 °C. To process for electron microscopy, tissue samples were osmicated using the ROTO staining method (reduced osmium tetroxide thiocarbohydrazide (TCH)-osmium). Sections were followed by incubations in an ascending ethanol series (5 minutes each in 50%, 70%, 80%, 90% ethanol/H_2_O), 2 minutes with 100% ethanol, overnight with 100% ethanol, 5 minutes with 50% propylene oxide (PO; EMS)/ethanol, 5 minutes with 75% PO/ethanol, 2 minutes with 100% PO (repeated 3 times), 1 hour with 50% epon based resin/PO, 4 hour with 75% epon based resin/PO, and overnight with 100% Epon based resin. Epon based resin were made by mixing 4.5 g of mix A (mixture of 10 g of LX112 and 10.9 g of Nonenyl Succinic Anhydride), 10.5 g of mix B (mixture of 18 g of LX112 and 15.47 g of Nadic Methyl Anhydride) and 0.1 ml of benzyl dimethylamine. LX112, Nonenyl Succinic Anhydride, Nadic Methyl Anhydride, and benzyl dimethylamine are purchased form LADD research industries. Curing of the resin was achieved at 65 °C for 2 days. Epon blocks were trimmed with a TrimTool diamond knife (Trim 45; DiATOME) and ultrathin sectioned with an Ultra Diamond Knife (Ultra 35°; DiATOME) in a Leica Ultramicrotome (UC7) and the serial sections collected using ATUM technology^24,39,40^. Approximately 1500 1 mm (width), 3 mm (length) 40 nm thick serial sections were obtained and collected by ATUM using carbon coated tape (kapton).

### Serial section electron microscopy

Placed in a silicon wafer, the region of interest (ROI) in the sections was identified by visualizing blood vessel land marks. Electron micrographs at higher resolution (4 nm pixel size, 1 µs dwell time) were obtained with a Sigma Field Emission Scanning Electron Microscope (7 KeV; Zeiss) using back-scatter signal detector. All image stacks obtained were then digitally processed, aligned and reconstructed in Fiji (NIH) using the TrackEM2 plugin^41^.

The time required for the procedures were 9 hours on the first day (from the fixation to DAB development), 3–4 hours on the second day (Osmium impregnation), 5–6 hours on the third day (Dehydration), 5 hours on the fourth day (Dehydration). After 2–3 days of embedding and 4–12 hours of sectioning and staining, imaging of the samples required up to one month and tracing required 1d to a month, depending on the thickness and resolution of the sample.

### Re-identification of AVC expressing neurons in ssEM

First, the cell body of the labeled neurons was identified by measuring the distance from the surface and ventricle of the neocortex, and sometimes using blood vessels as fiducials. Then, the axonal or dendritic segments were identified by measuring the distance from the cell body to the target regions and visual inspection of DAB staining. The shrinkage of the sample was negligible for identifying the segments of interest. The time spent for identifying the segments shown in the new Fig. 4 was less than 10 mins. For the axon shown in Fig. 5, the time spent was about 30 minutes to one hour since the axon is more torturous and required about 100 sections to be investigated.

## Electronic supplementary material

Supplementary Figure

Movie S1

Movie S2

Movie S3

Movie S4

Movie S5

Movie S6

Movie S7

Movie S8
