# Supplementary material for: Correlated Light-Serial Scanning Electron Microscopy (CoLSSEM) for ultrastructural visualization of single neurons in vivo
Source: Sci Rep. 2018 Sep 27;8:14491. doi: 10.1038/s41598-018-32820-5 (PMC6160427; doi:10.1038/s41598-018-32820-5)
Supplement: Supplementary file 1 — Supplementary Figure [file 41598_2018_32820_MOESM1_ESM.pdf]

Supplemental Figure

Correlated Light-Serial Scanning Electron Microscopy (CoLSSEM) for ultrastructural visualization of single neurons *in vivo*

Yusuke Hirabayashi [1,2,3,4], Juan Carlos Tapia [1,5] * and Franck Polleux [1,2,3] *

[1] Columbia University Medical Center - Department of Neuroscience

[2] Mortimer B. Zuckerman Mind Brain Behavior Institute

[3] Kavli Institute for Brain Science

[4] JST, PRESTO

550 W. 120th Street, Northwest Corner Building #1104, New York, NY 10027

[5] University of Talca, Talca, Chile – Department of Health Sciences

* Co-corresponding authors:

Franck Polleux, Ph.D.
Columbia University
Department of Neuroscience
Mortimer B. Zuckerman Mind Brain Behavior Institute
Kavli Institute for Brain Science

1108 NWC Building - MC 4862
550 West 120th Street
New York, N.Y. 10027
fp2304@cumc.columbia.edu

Tel: 212-853-0407

Juan Carlos Tapia, Ph.D.
University of Talca
Department of Health Sciences
Avda. Lircay S/N
Talca, Chile 3460000
juantapia@utalca.cl

Tel: 56-712-418855 extension 2855

Legends for supplementary figure and movies

Figure S1. Serial coronal vibratome slices (100 microns thick) of adult mouse brain following sparse, Cre-dependent, *in utero* cortical electroporation. Individual optically-isolated Venus-expressing pyramidal neurons in layer 2-3 were indicated with arrows. 15 neurons in this brain were suitable for CoLSSEM.

Figure S2. Tracing of non-labeled and labeled spines

Serial electron micrographs obtained with ATUM-SEM (A and B) and tracing of corresponding dendritic segment (A' and B') of unlabeled (A and A') and AVC(DAB)-labeled (B and B') dendritic segment. Arrowheads indicate discontinuous spine necks that in the Apex2-labeled segment helps disambiguate the small fragments of that spine (B-B').

Movie S1. Related to Figure 1E. Low magnification, 3D rotating projection of confocal stack of single cortical pyramidal neuron in layer 2/3 expressing CAAX-mVenus-Apex, imaged at P21.

Movie S2. Related to Figure 1E. High magnification and high resolution confocal imaging of a single dendritic branch of pyramidal neuron in layer 2/3 showing single dendritic spines.

Movie S3. Related to Figure 4. 3D reconstruction of the apical dendrite of a pyramidal neuron imaged by CoLSSEM, 3D SEM shown in Figure 4.

Movie S4. Related to Figure 5. Low magnification, 3D rotating projection of confocal stack of single cortical pyramidal neuron in layer 2/3 expressing CAAX-mVenus-Apex, imaged at P21. The axon segment of this neuron is imaged by 3D SEM in Figure 5.

Movie S5. Related to Figure 5. Raw image series used for 3D SEM of axon segment reconstructed in Figure 5.

Movie S6. Related to Figure 5. Annotated image series used for 3D SEM of axon segment reconstructed in Figure 5. The axon segment from neuron reconstructed in Figure 4 is outlined in blue.

Movie S7. Related to Figure 5. 3D animation of the axon segment imaged by CoLSSEM and traced in Figure 5 (also shown in Movies S5&S6).

Movie S8. Related to Figure 5. 3D animation of the axon segment (blue) imaged by CoLSSEM and traced in Figure 4 focusing on the presynaptic terminal labeled with the genetically encoded vGlut1-mCherry (inset of Figure 5A). The post-synaptic partner of this presynaptic bouton is shown in yellow.

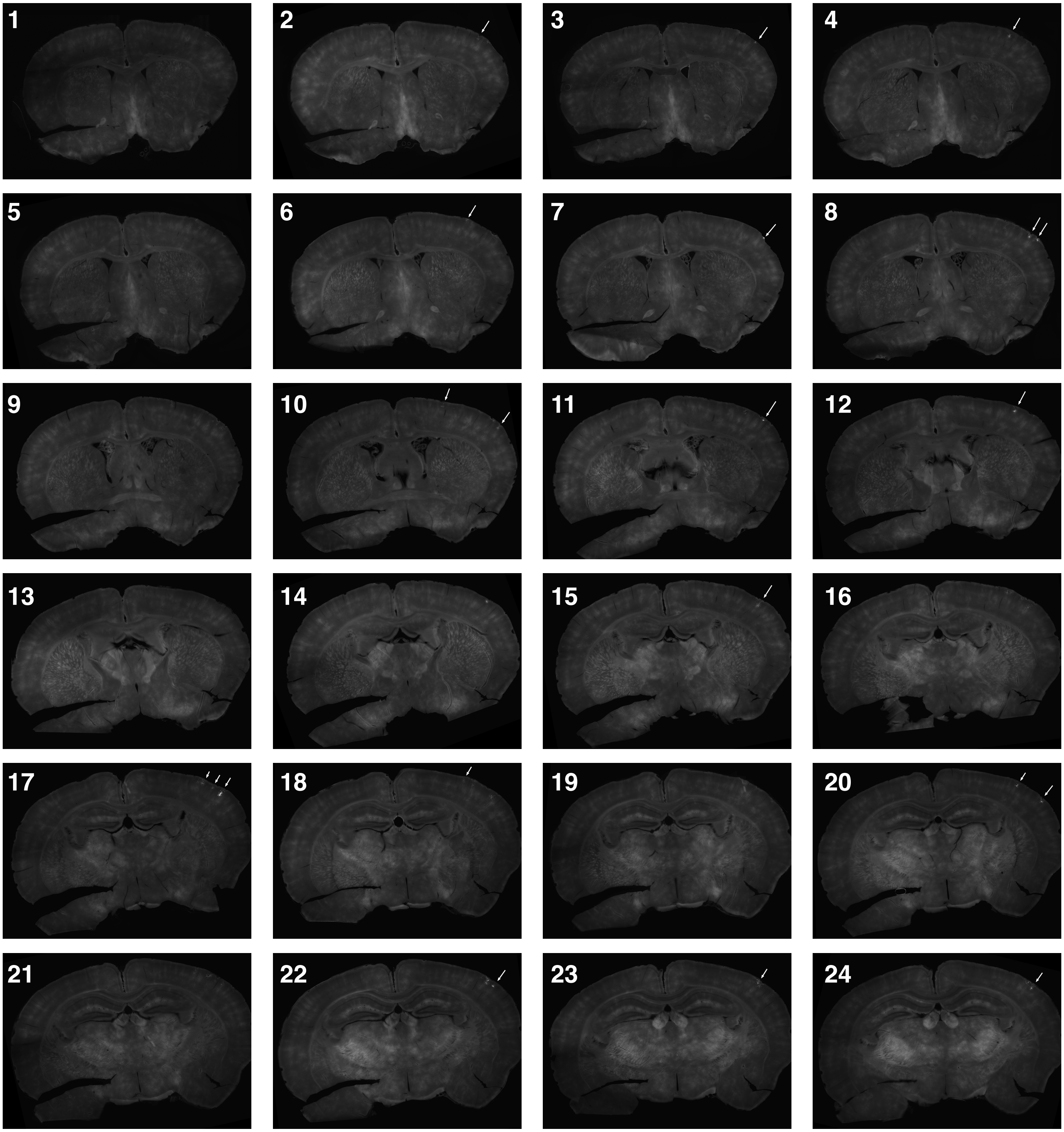

Supplemental Figure 1 - Hirabayashi et al.

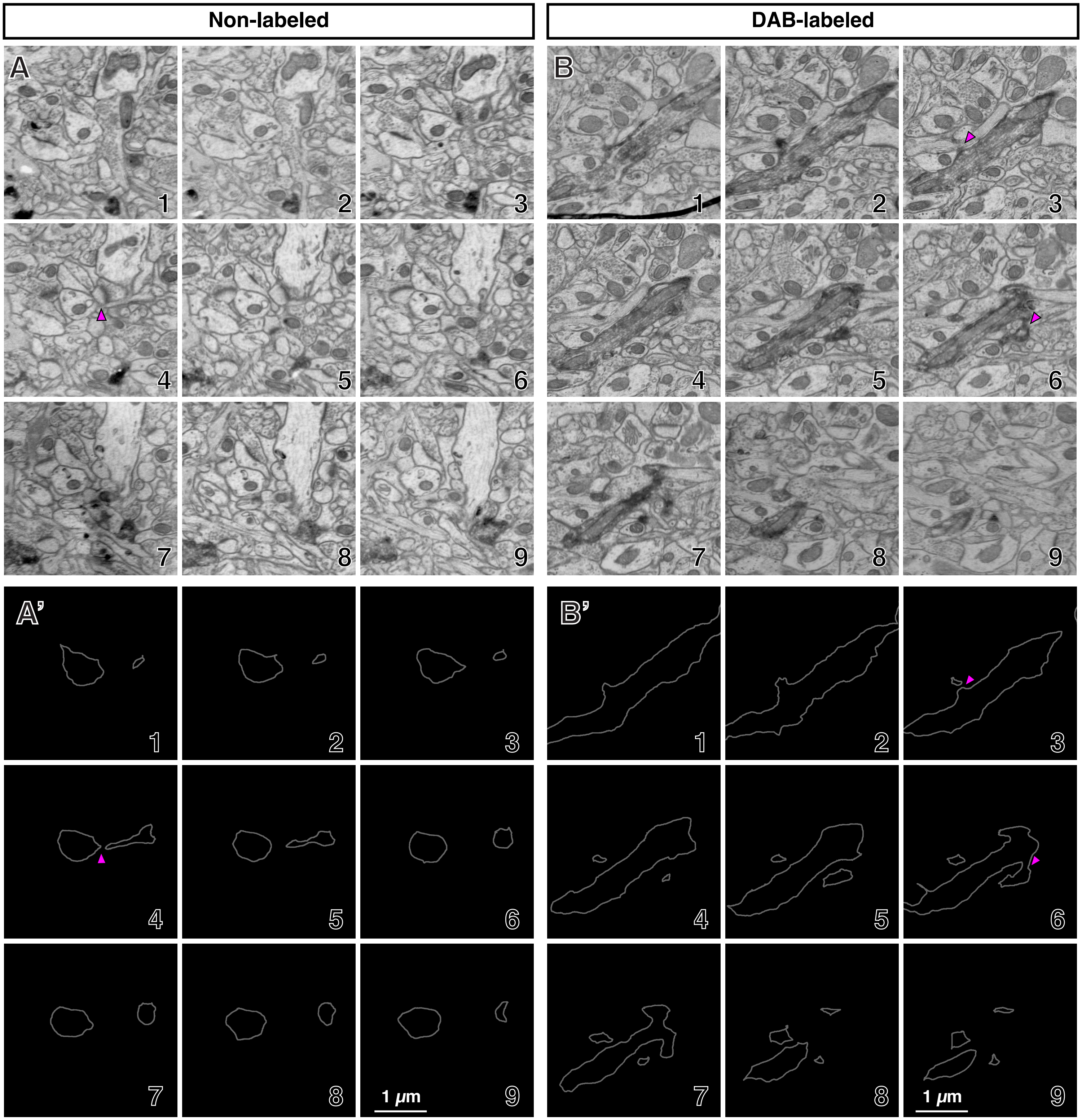

Supplemental Figure 2 - Hirabayashi et al.
